# Abstracts From the 9th Symposium in Metabolism

**DOI:** 10.1097/j.pbj.0000000000000037

**Published:** 2019-06-17

**Speakers**

**Excess adiposity and genitourinary cancers**

Ricardo Ribeiro

Tumor & Microenvironment Interactions Group, i3S Instituto de Investigação e Inovação em Saúde/INEB Instituto Nacional de Bioengenharia, Porto, Portugal, Genetics Laboratory and Environmental Health Institute, Faculty of Medicine, University of Lisbon, Portugal, Department of Clinical Pathology, Centro Hospitalar e Universitário de Coimbra, Coimbra, Portugal

Over the past years, increased interest has been devoted towards obesity-associated cancer. Genitourinary cancers are complex multi-factorial disease, characterized by intricate genetic and environmental interactions, ultimately leading to disease progression. The uniqueness of most common urological malignancies, prostate, urothelial and renal cancers, reveals interesting clues to our understanding of mechanisms underlying excess adiposity-cancer relationship, despite apparently paradoxical in some cases. The surrounding environment is likely to have a role, through provision of molecular and cellular signals that exert regulatory effects in tumor's critical pathways.

Recently added knowledge on molecular and functional profiling of adipose tissue type (white, brown and brite adipose tissue) and anatomical depots, revealed novel mechanistic insights to the association obesity-cancer and are clearing the fog away on our understanding of potential actionable pathways that might influence genitourinary cancer natural history. The obese setting provides a unique environment in adipose tissue with concomitant paracrine and endocrine alterations that influences tumor initiation and/or progression.

A series of recent basic and translational research studies uncovered previously unrecognized mechanisms behind the causally-related association between obesity and cancer, the adipose tissue-derived stem/stromal cells, epigenetic modifications, adipocyte-derived exosomes and miRNAs, besides the established influence of adipokines and chronic low-grade inflammation.

Taken together with epidemiological data showing a rise in obesity prevalence, accumulating evidence on mediators and mechanisms supporting an adipose tissue-cancer link, the time has come for including into clinical reasoning specific therapeutic strategies for patients with obesity that develop tumors. Therefore, our ability to treat cancer in obese patients will likely depend on how we effectively intervene in the pathways that link obesity to cancer.

Recognition of the mechanistic association between adipose tissue and specific particularities of genitourinary cancers may provide an opportunity for preventive and therapeutic strategies to reduce incidence, morbidity and mortality in the uro-oncological setting.

**The hepatic insulin/IGF1/PTEN signaling axis in metabolism and cancer**

Michelangelo Foti

Department of Cell Physiology and Metabolism, Diabetes Center, Faculty of Medicine, University of Geneva, Switzerland

Fatty liver disease (FLD) is tightly associated with obesity and diabetes. FLD initiates with the development of hepatic steatosis and insulin resistance, which can then progress towards inflammation and fibrosis. FLD is also a major risk factor for the development of hepatocellular carcinoma (HCC), which can occur in the presence or absence of cirrhosis. We previously showed that in humans and rodents, steatosis is associated with a significant downregulation of the expression/activity of PTEN, an important tumour suppressor downregulated, mutated or deleted in several cancers including HCC. PTEN is a lipid/protein phosphatase, which negatively regulates insulin receptor (IR) and IGF-1 receptor (IGF1R) signalling. IR and IGF1R are closely related tyrosine kinase receptors, which in response to their cognate ligands transduce signalling through the PI3K/AKT and MAPK pathways, albeit with different terminal outputs. Genetic studies investigating mouse phenotypes associated with the single deletion of each receptor have suggested that IR is involved in metabolic signaling whereas IGF1R triggers principally mitogenic signalling. In the liver, the PI3K/AKT pathway is a master regulator of the lipid and glucose metabolism, whereas alterations of both PI3K/AKT and MAPK signaling have been shown to contribute to carcinogenesis. PTEN antagonizes both PI3K and MAPK signalling thereby being able to regulate the metabolic activity of the liver and to act as a potent tumour suppressor. Consistent with this role, hepatocyte PTEN deficiency in mice (LPTENKO mice) triggers the development of fatty liver diseases and cancer with ageing.

These last years, we have developed complex genetic models of mice bearing hepatocytes-targeted deletions of PTEN and/or the IR/IGF1-receptors to understand how insulin and IGF-1 signalling in concert with PTEN regulate hepatic metabolic homeostasis and liver cancer development. Our studies showed that in the liver, signalling through IR and IGF1R, which are antagonized by PTEN, control distinct processes regulating hepatic glucose and lipid metabolism. More strikingly, hepatic IR and IGF1R signalling in concert with PTEN differentially impact on metabolic homeostasis of other peripheral organs (muscle, white and brown adipose tissues and pancreatic endocrine cells) through still poorly characterized crosstalk mechanisms. Finally, the role of IR and IGF1R signalling in the *in vivo* development of liver cancer is currently elusive. In this regard, our recent studies indicated that signalling through both receptors contribute to hepatic carcinogenesis. However, IR and IGF1R signalling have unexpected and distinct impacts on the type and malignancy of hepatic tumours induced by PTEN loss in hepatocytes.

Together, our studies outline the different contribution of hepatic IR and IGF1R signalling under the control of PTEN in metabolic homeostasis of the liver and peripheral organs, as well as in the development of hepatic cancers.

**Effect of polyphenols on nutrient cellular uptake: relevance for cancer and metabolic syndrome**

Fátima Martel

Unit of Biochemistry, Department of Biomedicine, Faculty of Medicine of Porto, and Instituto de Investigação e Inovação em Saúde (I3S), University of Porto, Portugal

Polyphenols, commonly contained in fruits and vegetables, have a beneficial action in the prevention of various diseases associated with oxidative stress, such as cancer and cardiovascular diseases. However, the actual molecular interactions of polyphenols with biological systems remain mostly unknown. Here, we present evidence that interference of polyphenols with nutrient cellular uptake is a mechanism contributing to their protective role against cancer and cardiovascular diseases/metabolic syndrome.

One of the cancer molecular hallmarks is a deviant energetic metabolism, known as the Warburg effect, whereby the rate of glucose uptake is significantly increased and a high rate of glycolysis and lactic acid production occurs even when oxygen is present—“aerobic lactatogenesis”. Accordingly, GLUT1 and MCT1, which are the main glucose and lactate transporters in cancer cells, respectively, were proposed as oncogenes and are currently seen as potential therapeutic targets in cancer treatment. *In vitro* studies have shown that several polyphenols act as specific inhibitors of glucose transport in breast cancer cell lines. Interestingly, an association between inhibition of glucose cellular uptake and their anticarcinogenic effect was found. Moreover, some polyphenols are able to inhibit lactate transport. Moreover, some polyphenols behave as inhibitors of both glucose and lactate cellular uptake by breast cancer cells, which is very interesting, because they deplete breast cancer cells of their two most important energy suppliers. So, the antimetabolic effect of polyphenols should be regarded as a mechanism of action contributing to their chemopreventive/chemotherapeutic potential in relation to cancer.

Maintenance of glucose homeostasis is of crucial importance to human physiology, and failure of this control can result in metabolic syndrome, a multifactorial condition leading to accelerated atherosclerosis and increased risk for diabetes, major cardiovascular events and a high mortality rate. Growing evidence indicates that various dietary polyphenols may influence glucose homeostasis at many levels, attenuating postprandial glycemic responses and fasting hyperglycemia, and improving acute insulin secretion and insulin sensitivity. The possible mechanisms include inhibition of carbohydrate digestion, stimulation of insulin secretion from pancreatic β-cells, modulation of hepatic glucose release and activation of insulin receptors and glucose uptake in insulin-sensitive tissues. Additionally, *in vitro* and *in vivo* inhibition by some polyphenols and polyphenol-rich foods of glucose intestinal absorption, through inhibition of the glucose transporters SGLT1 and/or GLUT2, has also been reported. Moreover, recent *in vitro* data show that some polyphenols are able to interfere with the intestinal absorption of fructose, which involves GLUT5 and GLUT2, a very interesting effect because intake of high-fructose products is associated with metabolic syndrome development. So, there is the potential for a variety of dietary polyphenols to affect intestinal glucose and fructose absorption *in vivo,* suggesting that foods and unsweetened beverages rich in these dietary polyphenols might provide a convenient dietary mechanism for regulating the rate of intestinal sugar absorption, an important factor in the management of diabetes, and in the long term might offer some protection against development of the metabolic syndrome, type 2 diabetes and cardiovascular disease.

**Cardiovascular disease and hepatic steatosis**

Elisabete Martins

Medicine Department, Faculty of Medicine, University of Porto, Cardiology Department, São João Hospital, Porto, Portugal, I3S- Institute for innovation and health research, University of Porto, Portugal

Nonalcoholic fatty liver disease (NAFLD) is an important cause of chronic hepatic disease and liver transplant in Western societies. The increasing prevalence is related to dietary changes and sedentarism and follows the increasing frequency of obesity and type 2 diabetes.

Recently, a growing evidence of the relation of NAFLD with cardiovascular diseases (CVD), independent of cardiovascular risk factors, has prompted the clarification of whether the liver is mainly a key-effector or a target-organ of the metabolic disarrangements in the metabolic syndrome. The therapeutic strategies able to alter liver disease progression and, through this, reduce the cardiovascular risk have also been tested in the last two decades.

This presentation focus on the possible interactions between hepatic disease, metabolic syndrome and CVD, and on their implications for clinical practice.

**Dietary cholesterol does not break your heart but kills your liver**

Gerhard P. Püschel

University of Potsdam, Institute of Nutritional Science, Germany

It is increasingly accepted that dietary cholesterol has a much lower impact on the progression of cardiovascular disease than previously assumed. However, both animal experiments and human studies seem to support the view that dietary cholesterol may contribute to the transition from benign hepatic steatosis to the potentially fatal NASH. Cholesterol esters and cholesterol accumulate in the hepatocyte and impair its function. This leads to oxidative stress and ER stress triggering the release of pro-inflammatory cytokines and rendering the hepatocyte more susceptible to apoptotic or necrotic cell death. Kupffer cells group around dying hepatocytes and phagocytose the hepatocyte debris and lipids. In addition, they are exposed to lipid peroxidation products released from hepatocytes. Kupffer cells, thus activated, release pro-inflammatory, chemotactic and profibrotic cytokines that promote inflammation and fibrosis. Therefore, dietary cholesterol may be harmful to the liver, in particular when administered in combination with polyunsaturated fatty acids that favor lipid peroxidation.

**Western diet versus Mediterranean diet in the development of metabolic syndrome, cardiovascular diseases and cancer**

Teresa F. Amaral

Faculty of Nutrition and Food Sciences of the University of Porto, Portugal

The available evidence concerning the effects of Western diet versus Mediterranean-like dietary patterns in the development of metabolic syndrome, cardiovascular diseases and cancers reveals a life course complex and relevant interrelationship. There is increasing knowledge on how nutrients, non-nutrient food components and whole diets may impact.

Observational studies have documented the association of Mediterranean-like dietary patterns with reduced cardiovascular disease incidence and mortality, lower rates of cancer and also all-cause mortality. Otherwise, Western dietary styles are consistently associated with antagonistic results. More recently, intervention studies have provided evidence on cardiovascular protection from a Mediterranean diet patterns in primary and secondary prevention of these outcomes.

Within this presentation, the large body of knowledge regarding the link between these dietary patterns and cardiovascular diseases and cancers will be outlined.

**Metabolic derangements and the failing heart: consensus and controversies**

Patrícia Lourenço

Serviço Medicina Interna do Centro Hospitalar de São João, Portugal

Heart failure (HF) is a leading cause of morbidity and mortality worldwide. HF is the final common pathway of many cardiovascular disorders and of an injured heart; ischemia, hypertension and valvular disorders are among the predominant contributors. The development of HF is not commonly dependent on primary alterations of cardiac metabolism, however, whatever the HF etiology; a metabolic dysregulation is a hallmark feature of HF. The failing heart can be seen as an “engine out of fuel”.

To better understand the metabolic derangements of the failing heart it is first necessary to revisit the metabolism of the normal heart. The heart consumes more energy than any other organ. The adult heart weights about 300 g but it pumps approximately 10 tons of blood and cycles about 6 kg of ATP every day. With little stored energy capacity, the heart requires a constant ATP synthesis. In order to do so, the human heart is like a metabolic omnivore, capable of utilizing virtually any available nutrient. Under normal conditions the healthy heart derives most of its energy from free fatty acid (FFA) oxidation (2/3 of energy production) with the other energy sources being glycolysis, glucose oxidation or lactate and ketone bodies utilization. FFA and glucose metabolism strictly regulate each other in a process known as the Randle Cycle. The normal heart has remarkable metabolic flexibility in response to its metabolic and physiologic environment, switching substrate preference according to the circumstances: FFA are the preferred energy source in the fasting state and non-stressful conditions and glucose is the preferred energy source in the fed state and stress conditions such as ischemia. This preference for glucose utilization in stress conditions occurs because it is a more efficient source of energy – the amount of ATP produced per O_2_ consumed is greater when glucose is oxidized than when FFA are oxidized.

Metabolic derangements in the failing heart are complex and not fully understood. Changes can occur in all components of cardiac energy metabolism: substrate uptake and utilization, oxidative phosphorylation and high-energy phosphate metabolism. HF courses with neurohumoral activation and sympathetic overdrive leading to alterations in substrate availability – elevation of plasma and intracellular FFA and insulin resistance – resembling metabolic syndrome alterations. In the early stages of HF it appears that FFA uptake and oxidation remains either unchanged or moderately increased and glucose oxidation is increased. As HF progresses excessive FFA metabolism can promote reactive oxygen species formation with impairment of mitochondrial and cardiac function. Eventually advanced stage HF emerges and both glucose and FFA oxidation are severely diminished and the heart becomes unable to transform chemical energy in mechanical energy of the actin-myosin interaction of myofibrils. Inability to use FFA and glucose in the setting of increased FFA and insulin resistance is the basis of lipotoxicity, glucotoxicity and glucolipotoxicity in which accumulation of substrates and intermediate metabolites further deteriorates mitochondrial function.

In metabolic syndrome a similar mismatch between substrates availability and oxidation is observed: the heart is flooded with substrates that exceed its capacity to use them and their accumulation as well as accumulation of intermediate metabolites will ultimately result in cardiomyocyte dysfunction.

HF may be dependent on and promote metabolic changes that yield a state of metabolic inefficiency. Modulation of cardiac metabolism holds promise as a new approach to HF treatment.

**The endothelial border to inflammatory and cardiovascular health**

Nina Wærling Hansen, Anker Jon Hansen, Anette Sams

Department of Clinical Experimental Research, Glostrup Research Institute, Copenhagen University Hospital Glostrup, Denmark

The endothelial cell layer constitutes a barrier that controls movements of fluid, solutes and cells between blood and tissue. Further, the endothelial layer regulates vascular tone and directs local humoral and cellular inflammatory processes. The strategic position makes it an important player for maintenance of health and for development of a number of diseases. Endothelial dysfunction is an important component of type 2 diabetes, and is also assumed to be involved in many other diseases, e.g., rheumatoid arthritis, inflammatory bowel disease, asthma, and cardiovascular diseases.

Various diseases involving inflammatory and immunological components are accelerated by hyperglycemic events because the endothelium transduces “high-glucose” signaling into significant pathophysiological phenomena leading to reduced endothelial barrier function, compromised vascular tone regulation and inflammation (e.g., cytokine secretion and RAGE activation). In addition, endothelial extracellular proteins form epitopes for potential specific antibody formation upon interactions with reducing sugars.

This presentation reviews the endothelial metabolism, biology, inflammatory processes, physical barrier functions, and summarizes evidence that although stochastic in nature, endothelial responses to hyperglycemia are major contributors to disease pathophysiology. We present molecular and mechanistic evidence that both biological and physical barriers, protein function, specific immunity, and inflammatory processes are compromised by hyperglycemic events and thus, hyperglycemic events alone should be considered risk factors for numerous human diseases.

**Short oral communications**

**Sirtuin 2 deficiency promotes exacerbated adiposity and hepatic lipid accumulation in mice subjected to diet-induced obesity**

M. Quatorze^1^, P. Valério^1^, H. Leal^1^, C. Cavadas^1,2^, P. Gomes^1^

^1^*Center for Neuroscience and Cell Biology (CNC), University of Coimbra, Coimbra, Portugal*, ^2^*Faculty of Pharmacy, University of Coimbra, Coimbra, Portugal*

The metabolic syndrome, which comprises excess body fat, insulin resistance, hypertension, glucose intolerance and dyslipidemia, is strongly associated with an elevated risk of developing cardiovascular diseases, diabetes, and cancer (1). The prevalence of these chronic pathologies is growing exponentially, which requires the development of novel therapeutic approaches to prevent or counteract their progression. The sirtuin family (SIRT1-7) of NAD^+^-dependent protein deacetylases have gained increased recognition as crucial regulators of metabolic homeostasis. SIRT2, one of the least understood sirtuin isoforms, has been studied, mostly in *in vitro* models, as a key player in various metabolic processes, such as adipogenesis, fatty acid oxidation and insulin sensitivity (2). However, the role of SIRT2 in the development of obesity and related metabolic abnormalities has not been explored in an *in vivo* mouse model. Therefore, the aim of this study was to provide novel insights into the role of SIRT2 in the metabolic changes mediated by diet-induced obesity, using a global SIRT2 knockout (SIRT2-KO) mouse model. Thus, it was performed a thorough metabolic characterization of SIRT2-KO mice fed a regular chow diet (CD) or a high-fat diet (HFD) for 4 weeks. Despite apparently normal under CD feeding, SIRT2-KO mice showed increased body weight gain, enlarged epididymal white adipose tissue mass, hypertriglyceridemia and hyperglycemia when fed a HFD. Moreover, these animals developed a phenotype of severe insulin resistance, although it was not observed changes in phosphorylation levels of intermediates of the insulin signaling cascade in the liver, adipose tissue and skeletal muscle after insulin challenge. SIRT2-KO mice fed a HFD exhibited enlarged adipocyte size and exacerbated lipid accumulation in the liver, when compared with wild-type animals. Lipid overload in the liver was consistent with upregulated gene expression of lipogenic enzymes. Our findings reveal that SIRT2 plays a crucial role in the liver and adipose tissue, although further studies are required to fully address SIRT2 metabolic actions. SIRT2 stimulation may be a therapeutic strategy to counteract obesity and associated metabolic complications.

**References**

1. Shulman, G. I., *N. Engl. J. Med.* 371.12:1131–1141, 2014.

2. Gomes, P., Outeiro, TF., Cavadas, C., *Trends Pharmacol. Sci.* 36.11:756–768, 2015.

**Acknowledgements**

Fundação para a Ciência e a Tecnologia (SFRH/BPD/111815/2015 to P.G.).

**The impact of exercise training in adipose tissue remodeling and cardiac dysfunction associated with obesity**

L. Costa^1^, N. Paiva^1^, R. Nogueira-Ferreira^2^, S. Magalhães^3^, R. Vitorino^2,3^, R. Ferreira^1^, D. Moreira-Gonçalves^2,4^

^1^*QOPNA, Department of Chemistry, University of Aveiro, Aveiro, Portugal*, ^2^*Departamento de Cirurgia e Fisiologia, Faculdade de Medicina, Universidade do Porto, Porto, Portugal*, ^3^*iBiMED, Department of Medical Sciences, University of Aveiro, Aveiro, Portugal*, ^*4*^*CIAFEL, Faculty of Sport, University of Porto, Porto, Portugal*

**Aim:** Our goal was to evaluate the molecular pathways modulated by exercise training in white adipose tissue and cardiac muscle in obesity, using an animal model of the disease.

**Introduction:** Obesity is considered a major health problem, being a significant risk factor for the development of a number of diseases such as cardiovascular diseases. Regular exercise training has been prescribed for weight loss, as a result of several changes in morphological and biochemical properties of white adipose tissue. On the other hand, exercise can also be cardioprotective as it improves cardiorespiratory function, by causing a decrease in blood pressure and myocardial fibrosis, and promotes physiological cardiac hypertrophy.

**Methods:** An animal model of obesity (ZSF1 rat) was submitted to 4-weeks of treadmill exercise training (5 days per week, 1 h per day, 15 m/min). Twenty-four hours after the end of the exercise training protocol, animals were anesthetized for hemodynamics analysis and then sacrificed through exsanguination. Left ventricle and visceral adipose tissue was excised. The levels of UCP-1, VEGF and oxidized (carbonylated and nitrated) proteins were determined in adipose tissue by immunoblotting. The levels of metalloproteases (MMP)-2 and -9 and its inhibitors (TIMPs) were assessed by western blotting in both samples. Zymography was also performed to evaluate proteases activity.

**Results:** Exercise training promoted the remodeling of adipose tissue in obese rats characterized by increased UCP-1 levels and protein nitration. MMP-2 and MMP-9 were not modulated in the adipose tissue by exercise training, unlike the initially expected considering its anti-inflammatory role. In cardiac muscle, the content of MMP-9 was responsive to 4-weeks of treadmill exercise but not its activity. So, MMPs activity does not seem to contribute to the exercise-related remodeling of adipose tissue or cardiac muscle in obese rats.

**Discussion/Conclusions:** Our results show evidences of adipose tissue remodeling promoted by exercise training in obese rats with benefic effects on cardiac functionality though without the apparent contribution of MMPs. Future work should focus on other molecular pathways modulated by inflammation in adipose tissue/cardiac muscle axis.

**Acknowledgments**

This work was supported by the European-Commission grant FP7-Health-2010; MEDIA-261409 and by Portuguese Foundation for Science and Technology (FCT), European Union, QREN, FEDER and COMPETE for funding the QOPNA (UID/QUI/00062/2013) and Unidade de Investigação Cardiovascular (UID/IC/00051/2013).

**Prevalence and risk factors for death by cardiovascular, infection or cancer complications in a diabetic food cohort**

J. Rigor^1^, S. Pinto^1^, D. Martins-Mendes^1,2,3^, Consulta Multidisciplinar de Pé Diabético do CHVNG/Espinho EPE^1^, M. Monteiro-Soares^4,5^

^1^*Centro Hospitalar de Vila Nova de Gaia/Espinho EPE*, ^2^*Departamento de Bioquímica da Faculdade de Medicina da Universidade do Porto*, ^3^*I3S - Instituto de Investigação e Inovação da Universidade do Porto*, ^4^*MEDCIDS - Departamento de Medicina da Comunidade, Informação e Decisão em Saúde da Faculdade de Medicina da Universidade do Porto*, ^5^*CINTESIS - Centro de Investigação em Tecnologias e Serviços de Saúde*

**Introduction:** The metabolic syndrome highly increases the risk of Diabetes mellitus (DM), which can lead to several complications. Diabetic foot ulcers (DFU) are responsible for a high level of morbidity and mortality. DFUs are responsible and/or a marker of inflammatory, metabolic and vascular complications. We aim to quantify and determine which patients are at higher risk of death due to cardiovascular, infection or cancer complications.

**Methods:** We conducted a cohort study with consecutive inclusion of individuals with an active DFU that were followed in our Diabetic Foot Clinic from January 2010 to March 2013. At baseline, demographic and clinical characterization variables were collected. Consulting participants’ clinical files and the National Data Platform, a 3 year follow up was performed. We analysed the prevalence and association, using univariate analysis methods, between the collected variables and the risk of dying due to cardiovascular, infection or cancer complications.

**Results:** A total of 293 participants were included, with a mean age of 68 years, DM duration of 18 years, HbA1c of 8.1% and body mass index (BMI) of 27 kg/m^2^. During follow-up, 30% of the individuals died (n = 89). Median time until death was 22 months. The most frequent causes were infection (50%), cardiovascular disease (24%) and cancer (10%). Variables associated with death by cardiovascular complications were BMI, insulin use and retinopathy. Variables associated with death by infection were age, physical impairment, end-stage renal disease, previous DFU. Variables associated with death by cancer complications were BMI, retinopathy and diabetic peripheral neuropathy.

**Conclusions:** Individuals with a DFU history have an important risk of dying at short term. Infection susceptibility, cardiovascular and malignant diseases are the most frequent causes. The mechanisms related to this clinical outcome should be further studied and preventive measures proposed and implemented.

**Financial support:** Matilde Monteiro-Soares work was financed by Project “NORTE-01-0145-FEDER-000016” (NanoSTIMA) that is financed by the North Portugal Regional Operational Programme (NORTE 2020), under the PORTUGAL 2020 Partnership Agreement, and through the European Regional Development Fund (ERDF).

**Metformin induces an increase in glucose uptake and lactate production by breast cancer cells**

I. Amaral^1,2^, C. Silva^1,2^, A. Correia-Branco^1,2^, F. Martel^1,2^

^1^*Department of Biomedicine – Unit of Biochemistry, Faculty of Medicine of Porto, University of Porto, Portugal*, ^2^*i3S – Instituto de Investigação e Inovação em Saúde, University of Porto, Portugal*

Breast cancer is a major global health burden [1]. Nutrient uptake and metabolism, more specifically glucose uptake and metabolism, is a newly recognized cancer therapeutic target. Metformin, an anti-diabetic drug, has been widely tested for its anticarcinogenic potential, including metabolic modulation characteristics [2]. This work aims to investigate the effect of metformin on glucose uptake and metabolism, by breast cancer cells, as a mechanism contributing to its anticancer properties.

Estrogen and progesterone receptor-positive (MCF-7) and triple-negative (MDA-MB-231) breast cancer cell lines were used as in-vitro models of breast cancer. Transport experiments with ^3^H-deoxy-D-glucose (^3^H-DG) and assessment of glucose glycolytic metabolism by lactate production was done. Culture mass was quantified with the sulforhodamine B assay (SRB), cell proliferation rate was determined by measuring ^3^H-thymidine incorporation and cell viability was evaluated by the MTT assay.

Acute (26 min) exposure of MCF-7 cells to metformin significantly inhibited uptake of ^3^H-DG (maximal inhibition found with metformin 0.5 mM: 27 ± 2% reduction). Chronically (24 h), metformin induced a concentration-dependent increase in ^3^H-DG uptake (maximal increase observed with metformin 1 mM: 81 ± 15% increase). Acute (26 min) exposure of MDA-MB-231 cells to metformin slightly inhibited uptake of ^3^H-DG (maximal inhibition found with metformin 1 mM: 10 ± 3% reduction). Chronic (24 h) exposure to metformin significantly increased ^3^H-DG uptake by MDA-MB-231 cells (maximal increase observed with metformin 1 mM: 30 ± 8% increase). Chronic (24 h) exposure to metformin (1 mM) significantly increased lactate production in both MCF-7 (to 467 ± 59% of control) and MDA-MB-231 (to 121 ± 3% of control) cell lines. Chronic (24 h) exposure of MCF-7 and MDA-MB-231 cells to metformin reduced cell viability and culture mass in a concentration-dependent manner; in contrast, it slightly increased cell proliferation rates. Combination of metformin (1 mM) with the facilitative glucose transporter (GLUT) inhibitor kaempferol (30 mM) did not result in a more marked effect on culture mass and cell proliferation rates.

It is known that metformin inhibits oxidative phosphorylation by inhibiting mitochondrial complex I, leading to a compensatory increase in glycolytic ATP and lactate production [2]. Therefore, we hypothesize that acutely, metformin reduces glucose uptake by directly inhibiting GLUT. However, chronically, with constant inhibition of mitochondrial complex I by metformin, with a resultant ATP deficiency state, the cells try to surpass the energetic deficiency by increasing glucose cellular uptake (either by placing more GLUTs in the membrane or by increasing its intrinsic activity) and the glycolytic production of lactate. We thus suggest that the increase in glucose uptake is a compensatory mechanism to cellular energy depletion induced by metformin. Metformin-induced dependence of breast cancer cells on glycolytic pathway, associated with an anticarcinogenic effect of the drug, provides a biochemical basis for the design of new therapeutic strategies.

**References**

1. DeSantis et al. CA Cancer J. Clin. 64 (2014) 52–62.

2. Jara, López-Muñoz. Pharmacol Res. 101 (2015) 102–108.

**Funding:** This study was supported by Instituto de Investigação e Inovação em Saúde, Universidade do Porto, Portugal (Plano estratégico UID/BIM/04293/2013).

**Metabolic syndrome after acute coronary syndrome: prevalence and impact of cardiovascular rehabilitation program**

H. Amorim^1^, R. Cadilha^1^, A. Rocha^1^, F. Parada^1^

^1^*Department of Physical and Rehabilitation Medicine, Centro Hospitalar São João, Portugal*

**Introduction:** Metabolic syndrome (MSy) represents a cluster of obesity-related vascular risk factors and has been associated with development and progression of coronary heart disease (CHD). Prevalence amongst CHD engaged in cardiac rehabilitation program (CRP) is variable and can reach 50%. Influence of MSy over patient's response to CRP remains to be elucidated.

We aimed to assess the prevalence of MSy in patients admitted to CRP after an acute coronary syndrome (ACS) and compare clinical and functional response between those with and those without MSy.

**Methods:** We conducted a retrospective study of prospectively collected data in patients consecutively referred to a CRP after ACS between September of 2008 and July of 2017. Clinical data, laboratory tests, and exercise testing results abstracted from electronic clinical records. The CRP included dietary counseling, and exercise training intervention consisting of 8–16 twice-weekly sessions, lasting 45 to 60 minutes, including aerobic and resistance training. MSy was defined at hospital admission according to International Diabetes Federation criteria. We used means and standard deviation for continuous variables, proportions for categorical variables. For between-group comparison (MSy vs non-MSY), we used the Qui^2^ test for categorical and T-student test for continuous variables.

**Results:** 922 patients were admitted during this period, 785 (85.1%) were men, with a mean age of 54.7 ± 10.1 years. Of these patients 257 (27.9%) fulfilled criteria for MSy, 219 (85.2%) of those were men with mean age of 54.6 ± 10,1. SMy patients had more frequently non-ST segment elevation ACS (SMy: 16.0%; NSMy: 11.0%; p < 0.05), multiple coronary vessels disease (SMy: 45.3%; NSMy: 34.2%; p < 0.01), and previous history of coronary event (SMy: 21.5%; NSMy:12.1%; p < 0.01), reflecting a longer and more diffuse CHD. In addition, they showed worse functional capacity results at baseline exercise testing in both duration (mean difference (MD): −43 ± 10 seconds; p < 0.01) and intensity in metabolic equivalents (METS) (−0.78 ± 0.18 METS; p < 0.01). There were no differences between groups regarding age, sex, left ventricular function, smoking status, adherence to the CRP, quality of life and previous physical activity. After CRP, only SMy patients had a significant weight loss (MD: −1.38 ± 5.75 Kg; p < 0.01) compared to NSMy (−0.56 ± 27.1 Kg; p = 0.63). Both groups had a significant decrease in waist circumference, with higher absolute reduction in SMy (- 2.49 ± 2.99 cm; p < 0.01) vs NSMy (- 1.66 ± 3.55 cm; p < 0.01). Both groups improved significantly (p < 0.05) in other cardiovascular risk factors including systolic/diastolic hypertension and triglycerides, although HDL cholesterol only improved in the NMSy group and fasting blood glucose did not change significantly in any of the groups during the CRP (p > 0.05). Both groups also improved their functional capacity (p < 0.01) and quality of life (p < 0.01) after conclusion of the program.

**Conclusions:** MSy was prevalent in our sample in similar proportion to what is estimated in the general adult European population. MSy patients who took part in a CRP had a more diffuse coronary disease and lower functional capacity. CRP after ACS benefits MSY patients, providing significant improvements in cardiovascular risk profile, functional capacity and quality of life.

**Funding:** None to report.

**Unravelling the regulation of biological processes by polyphenols in the cardiovascular system: a bioinformatics approach**

A. Rachão^∗1^, A.F. Silva^∗2,3^, R. Nogueira-Ferreira^2^, F. Trindade^2,3,4^, R. Vitorino^2,3,4^, A. Leite-Moreira^2,3^, D. Moreira-Gonçalves^3,5^, T. Henriques-Coelho^2,6^, R. Negrão^1,7^

^1^*Departamento de Biomedicina – Unidade de Bioquímica, Faculdade de Medicina da Universidade do Porto, Portugal,* ^2^*Unidade de Investigação Cardiovascular, Faculdade de Medicina da Universidade do Porto, Portugal,* ^3^*Departamento de Cirurgia e Fisiologia, Faculdade de Medicina da Universidade do Porto, Portugal,* ^*4*^*Instituto de Biomedicina - iBiMED, Departamento de Ciências Médicas, Universidade de Aveiro, Portugal*, ^*5*^*Centro de Investigação em Atividade Física, Saúde e Lazer, Faculdade de Desporto da Universidade do Porto, Portugal,* ^*6*^*Departamento de Ginecologia, Obstetrícia e Pediatria, Faculdade de Medicina da Universidade do Porto, Portugal,* ^*7*^*I3S-Instituto de Investigação e Inovação em Saúde, Universidade do Porto, Portugal*

^∗^Augusto Rachão and Ana Filipa Silva contributed equally to this work

**Introduction:** CVD stand as a great cause of morbi-mortality worldwide and polyphenol-rich diets have been associated with improved cardiovascular risk profiles. Although rodent models have been a resourceful mean of understanding the CVD mechanisms and possible outcomes of the use of polyphenols in that context, most experimental models do not fully reproduce human CVD.

**Aim:** To evaluate the cardiovascular system-related biological processes (BP) modulated by polyphenols in rodents and humans, and to verify which of them are specie-specific, in order to understand which outcomes for cardiovascular diseases (CVD) could be translated from animal to human studies.

**Methods:** Database searching was carried out on PubMed and Google Scholar using specific keywords concerning CVD, retrieving close to 300 publications. After excluding irrelevant results, proteome data was organized in Excel^®^ spreadsheets and the Cytoscape platform, ClueGo+CluePedia and Venny 2.1.0 were used to explore the biological processes influenced by flavonoids in the approached CVD.

**Results:** This study was mainly focused in the species *Rattus norvegicus* and *Homo sapiens* and in flavonoids, a polyphenol subgroup. Only about 5% of the BP influenced by flavonoids were common to both species and they were mostly related to the maintenance of blood pressure and the fatty acid metabolic process. Nevertheless, these effects were accomplished through different proteins/pathways and different subgroups of flavonoids.

**Discussion/Conclusion:** Our research highlights the need of careful translation of the flavonoids’ effects observed in rat models to clinical trials, since different proteins and subgroups of flavonoids mediated the observed actions. Though this type of studies can provide insights to help choosing the most adequate polyphenols as preventive approaches or therapies for human CVD, further investigation should be performed to clarify the described effects. Besides, pharmacokinetic aspects of the flavonoids’ action should also be considered when planning clinical trials.

**Acknowledgements**

This work was supported by Portuguese Foundation for Science and Technology grants PEst-OE/SAU/UI0038/2014; UID/BIM/04293/2013, UID/IC/00051/2013 (financed by Fundo Europeu do Desenvolvimento Regional through COMPETE 2020 – Programa Operacional Competitividade e Internacionalização) and The European Foundation for Alcohol Research (ERAB) (EA 14 23).

**Posters**

**Vitamin D nutritional status in individuals with obesity classified as metabolically unhealthy**

A. Cordeiro^1,2^, S.E. Pereira^1^, B. Campos^3^, C.J. Saboya^3^, A. Ramalho^1^

^1^*Department of Social Applied Nutrition, Micronutrients Research Center (NPqM), Institute of Nutrition, Federal University of Rio de Janeiro (UFRJ), Rio de Janeiro, Brazil*, ^2^*Department of Biomedicine, Unit of Biochemistry, Faculty of Medicine of the University of Porto (FMUP), Porto, Portugal,* ^3^*Multidisciplinary Center of Bariatric and Metabolic Surgery Carlos Saboya, Rio de Janeiro, Brazil*

**Background:** The prevalence of obesity has been increasing exponentially in recent years and it is the fifth greatest risk factor for mortality worldwide. Although obesity is the major starting factor for metabolic complications, there is a group of individuals with obesity that appear to be more protected from metabolic disorders and these are called as metabolically healthy obesity (MHO). Vitamin D (25OH)D acts in a various homeostatic processes, besides development and maintenance of bone tissue, according to the expression of its receptor in various cell types. It is a nutrient with an important role in obesity and associated diseases, may be engaged in multiple cellular processes, including the response to insulin.

**Objective:** To evaluate 25 (OH)D serum concentrations in MHO and metabolically unhealthy obesity (MUHO) and its relation with biochemical and clinical parameters in these both groups according to Homeostatic model assessment - insulin resistance (HOMA-IR) definition of the obesity phenotypes.

**Methods:** A descriptive cross-sectional study was conducted with individuals of both sexes, aged 21 - 62 years. Anthropometric data [waist circumference (WC), besides BMI] and metabolic parameters [blood pressure and blood glucose, glycated haemoglobin, insulin, lipid profile, calcium, phosphorus, parathyroid hormone (PTH) and high-sensitivity c- reactive protein (hs-CRP) and vitamin D [(25 (OH)D)] were obtained. The cut-off points for vitamin D deficiency and insufficiency were ≤20 and 21–29 ng/mL, respectively. Individuals were classified as MUHO according to HOMA-IR ≥ 2.5.

**Results:** This study comprised 232 individuals with obesity [body mass index (BMI) ≥ 35 Kg/m^2^; 42.6 ± 4.7 Kg/m^2^], 178 female (76.7%). The MUHO phenotype was observed in 76.7% of the population. The mean values of glucose (*p* < 0.001), insulin (*p* < 0.001), HOMA-IR (*p* < 0.001) and triglycerides (*p* = 0.049) were significantly higher in the MUHO than in the MHO phenotype group. The mean value of 25 (OH)D showed a significant difference between the MHO and MUHO phenotype groups (*p* = 0.011). Additionally, and in line, lower mean 25 (OH)D values were found in the MUHO versus the MHO phenotype group in the deficiency (14.5 ± 3.6 ng/mL/17.1 ± 2.7 ng/mL, *p* = 0.004) and insufficiency (24.5 ± 2.9 ng/mL/25.7 ± 2.6 ng/mL, *p* = 0.077) 25 (OH)D groups. An increase of 1 ng/mL of vitamin D increased in 1.051 [(95%CI: 1.011–1.093), *p* = 0.012] the odds of the healthy phenotype.

**Conclusion:** The highest prevalence of inadequacy of serum concentrations of 25 (OH)D and the greater severity of this deficiency were observed in individuals with MUHO phenotype. Low serum concentrations of this vitamin were associated, mainly, with IR. Monitoring the nutritional status of VD in individuals with obesity that present MUHO phenotype may contribute to minimize the occurrence and aggravation of diseases associated with obesity.

**Serum concentration of vitamin A and its relation with body adiposity, oxidative stress and cardiovascular risk in women with recommended dietary intake of vitamin A**

C. Bento^1^, A. Cordeiro^2,3^, A.C. Matos^2^, A. Ramalho^2^

^1^*Dietetic and Nutrition Department, Institute of Nutrition Josué de Castro (INJC) of the Federal University of Rio de Janeiro (UFRJ), Rio de Janeiro, Brazil,* ^2^*Department of Social Applied Nutrition, Micronutrients Research Center (NPqM), Institute of Nutrition, Federal University of Rio de Janeiro (UFRJ), Rio de Janeiro, Brazil,* ^3^*Department of Biomedicine, Unit of Biochemistry, Faculty of Medicine of the University of Porto (FMUP), Porto, Portugal*

**Background:** Evidence indicates the association of vitamin A in the regulation of fat mass demonstrating that vitamin A deficiency (VAD) brings about increase in the recruitment of pre-adipocytes to adipocytes, inhibition of apoptosis, and reduction in the adaptive thermogenesis, which influences on obesity and cardiovascular diseases.

**Material and Methods:** Cross-sectional study with 200 women, paired by age and dietary intake of vitamin A. Participants were divided into four groups, according to body mass index (BMI): 80 eutrophic (E), 40 overweight (OW), 40 class I obesity (OI) and 40 class II obesity (OII). Lipid and glycemic profiles were measured and oxidative stress was evaluated through serum concentrations of uric acid, glutathione peroxidase (GSH-Px), and thiobarbituric acid reactive substances (TBARS). The cutoff points for deficiency of serum concentrations of retinol and β-carotene were < 1.05 μmol/L and 40 μg/dL, respectively. The recommended dietary intake of vitamin A was 700 μg/day.

**Results:** Retinol and β-carotene deficiency was found in E group, 5% and 15%, respectively, reaching 77.5% and 82.5% in OII. Significant correlation between serum concentrations of retinol and biochemical markers of oxidative stress was presented in all groups, and as BMI increased the correlation was greater [OW – Retinol x GSH-Px r = 0.80; uric acid r =  − 0.78; TBARS r =  − 0.91//OII – Retinol x GSH-Px r = 0.86; uric acid r =  − 0.89; TBARS r =  − 0.92 (*p* = 0.001)]. It was observed that women in OI and OII groups who had retinol and β-carotene deficiency presented a risk for diagnosis of type 2 diabetes mellitus 16 and 20.7 times greater, respectively, when compared to E group with adequate concentrations of vitamin A.

**Conclusions:** The increased demand of vitamin A may be related to increase in BMI, body adiposity and oxidative stress, even when the recommended intake of vitamin A was reached.

**Age at menarche and development of metabolically healthy and unhealthy phenotypes in adolescents**

A.C.L. Magalhães^1^, A. Cordeiro^1,2^, M.N.G. Oliveira^3^, A.P.T. Pierucci^1^, P.C. Jesus^1^, A. Ramalho^1^

^1^*Department of Social Applied Nutrition, Micronutrients Research Center (NPqM), Institute of Nutrition, Federal University of Rio de Janeiro (UFRJ), Rio de Janeiro, Brazil,* ^2^*Department of Biomedicine, Unit of Biochemistry, Faculty of Medicine of the University of Porto (FMUP), Porto, Portugal,* ^3^*Adolescent Reference Center*

**Introduction:** Age at menarche is considered an important factor associated with development of metabolic syndrome (MS) and cardiovascular disease (CVD) in adulthood^1^. At present, it has been discussed about the appearance of metabolically healthy (MH) and unhealthy (MUH) phenotypes^2^, however, there are no studies which analyze the association between time of first menstruation and these metabolic profiles during adolescence.

**Objective:** To analyze the association between age of menarche and factors related to the development of MH and MUH phenotypes in adolescents.

**Methods:** Observational and cross-sectional study of adolescents of the Adolescent Reference Center, a multidisciplinary clinic located in the city of Macaé, Rio de Janeiro. The variables sexual maturation and age of the menarche were obtained through declaration of each participant, and was considered early menarche (EM) when the first menstruation occurred until 11 years old, normal menarche (NM) between 12 and 14 years, and late menarche (LM) after 15 years. For the analysis of the body variables, waist circumference (WC), weight, height and body mass index (BMI) were assessed. Blood pressure was measured and serum concentrations of HDL-c, triglycerides, fasting glucose and leptin were evaluated. The cut-off of leptin concentrations ≥ 11.6 ng/mL was considered inadequacy, as suggested in ELISA DBC Inc kit. Adolescents were classified in MH and MUH according to the criteria established by the NCEP-ATP III^3^, adapted for children and adolescents.

**Results:** One hundred thirty-nine adolescents participated in the study, 18% were MUH. When analyzing the association between the phases of sexual maturation and the metabolic profile, it was observed a higher prevalence of MUH in the pubertal phase of sexual maturation of pubic hair (*p* = 0.05) and breasts (*p* = 0.00). Regarding the age of menarche, it was noticed a higher prevalence of EM in the MUH group (n = 15; *p* = 0.04), of NM in MH (n = 71; *p* = 0.04) and none had LM. When evaluating the association between BMI and metabolic phenotype, it was noted that 8.1% of eutrophic adolescents were MUH (*p* = 0.00) and 29.4% with severe obesity were MH (*p* = 0.00). Highest mean of serum leptin concentrations was observed in adolescents with severe obesity who were MUH (32.93 ± 28.85 ng/mL; *p* = 0.01) and presented EM (22.90 ± 16.84 ng/mL; *p* = 0.05).

**Conclusion:** EM represents a risk factor for development of MUH phenotype, mainly due to the high BMI and highest mean of serum leptin concentrations. Thus, adolescents who mature early may be more susceptible to the risk of MS and CVD in adulthood.

**References**

1. STOCKL, D. et al. Age at menarche and its association with the metabolic syndrome and its components: Results from the KORA F4 study. PLoS ONE, v.6, n.10, p. E26076, 2011.

2. KARELIS, A.D. et al. Metabolic and body composition factors in subgroups of obesity: what do we know? The Journal of Clinical Endocrinology and Metabolism, v.89, n.6, p.2569–2575, 2004.

3. Executive summary of the third report of the third report of The National Cholesterol Education Program (NCEP) Expert panel on detection, evaluation, and treatment of high blood cholesterol in adults (Adult treatment panel III).JAMA, v.285, n.19, p.2486–2497, 2001.

**Sponsorship:** Fundação de Amparo à Pesquisa do Estado do Rio de Janeiro – FAPERJ.

**The NAD^+^-dependent deacetylase SIRT2 attenuates oxidative stress and mitochondrial dysfunction and improves insulin sensitivity in hepatocytes**

V. Lemos^1^, R.M. Oliveira^2^, L. Naia^3^, E. Szego^4^, E. Ramos^5,6^, S. Pinho^7^, F. Magro^1^, C. Cavadas^3,8^, A.C. Rego^3,9^, T. Costa^10,11,12^, T.F. Outeiro^2,4^, P. Gomes^1,3^

^1^*Department of Biomedicine, Faculty of Medicine, University of Porto, Porto, Portugal,* ^2^*Chronic Diseases Research Center (CEDOC), Faculty of Medical Sciences, Nova University of Lisbon, Lisbon, Portugal,* ^3^*Center for Neuroscience and Cell Biology (CNC), University of Coimbra, Coimbra, Portugal,* ^*4*^*Department of Experimental Neurodegeneration, Center for Biostructural Imaging of Neurodegeneration, University Medical Center Göttingen, Göttingen, Germany,* ^*5*^*Department of Clinical Epidemiology, Predictive Medicine and Public Health, Faculty of Medicine, University of Porto, Porto, Portugal,* ^*6*^*Department of Public Health Nutrition, Institute of Public Health of the University of Porto (ISPUP), Porto, Portugal,* ^*7*^*Department of Physiology and Cardiothoracic Surgery, Faculty of Medicine, University of Porto, Porto, Portugal,* ^*8*^*Faculty of Pharmacy, University of Coimbra, Coimbra, Portugal,* ^*9*^*Faculty of Medicine, University of Coimbra, Coimbra, Portugal,* ^*10*^*Instituto de Investigação e Inovação em Saúde (i3S), University of Porto, Porto, Portugal,* ^11^*Instituto de Biologia Molecular e Celular (IBMC), University of Porto, Porto, Portugal,* ^12^*Instituto de Ciências Biomédicas Abel Salazar, University of Porto, Porto, Portugal*

Insulin resistance is a major predictor of the development of metabolic disorders. Sirtuins (SIRTs) have emerged as potential targets that can be manipulated to counteract age-related diseases, including type 2 diabetes. SIRT2 has been recently shown to exert important metabolic effects (Gomes et al., *Trends Pharmacol Sci*, 2015), but whether SIRT2 regulates insulin sensitivity in hepatocytes is currently unknown. The aim of this study is to investigate this possibility and to elucidate underlying molecular mechanisms. Here we show that SIRT2 is downregulated in insulin-resistant hepatocytes and livers and this was accompanied by increased generation of reactive oxygen species (ROS), activation of stress-sensitive ERK1/2 kinase, and mitochondrial dysfunction. Conversely, SIRT2 overexpression in insulin-resistant hepatocytes improved insulin sensitivity, mitigated ROS production and ameliorated mitochondrial dysfunction. Further analysis revealed a reestablishment of mitochondrial morphology, with a higher number of elongated mitochondria rather than fragmented mitochondria instigated by insulin resistance. Mechanistically, SIRT2 was able to increase fusion-related protein Mfn2 and decrease mitochondrial-associated Drp1. SIRT2 also attenuated the downregulation of TFAM, a key mtDNA-associated protein, contributing to the increase in mitochondrial mass. Importantly, we found that SIRT2 expression in PBMCs of human subjects was negatively correlated with obesity and insulin resistance. These results suggest a novel function for hepatic SIRT2 in the regulation of insulin sensitivity and raise the possibility that SIRT2 activators may offer novel opportunities for preventing or treating insulin resistance and type 2 diabetes.

**Acknowledgements**

Fundação para a Ciência e a Tecnologia (SFRH/BPD/111815/2015 to P.G.).

**Frequency of metabolic syndrome in women with fibromyalgia and sedentary behavior**

A.C.K. Titski^1,2^, J. Mota^2^, D. Homann^1^, K. Zwiener^1^, N. Leite^1,2^

^1^*Federal University of Paraná – Curitiba – Brazil,* ^2^*Porto University – Porto - Portugal*

**Objective:** To compare the frequency of metabolic syndrome in women with fibromyalgia (patients) and without fibromyalgia (controls), with sedentary behavior.

**Methods:** 39 patients and 32 controls, aged between 20 and 50 years, participated in the study. Weight, height, body mass index (BMI), waist circumference (CA) and systolic (SBP) and diastolic (DBP) blood pressure were evaluated. The blood variables measured were glycemia (GLI), high-density lipoprotein (HDL-C) and triglycerides (TG). International Physical Activity Questionary (IPAQ) was applied, considering behavior sedentary as < 150 minutes/week. Metabolic syndrome was classified according to the criteria proposed by the Third Report of the National Cholesterol Education Program Expert Panel on Detection, Evaluation and Treatment of High Blood Cholesterol in Adults (NCEP-ATP III), with three or more of the following criteria: CA> 88 cm; PAS and/or PAD≥130/85mmHg; HDL-c < 50 mg/dL; TG≥150 mg/dL and GLI≥100 mg/dL. In the statistical analysis, the chi-square test was used, considering p < 0.05.

**Results:** Overweight was observed in 61.54% (n = 24) of the patients and in 56.24% (n = 18) of the control group (x2 = 0.053, p = 0.9787), while the metabolic syndrome was present in 28.2% of the patients and 6.25% in the women without fibromyalgia (x2 = 5.665; p = 0.038). In the group of patients, the inadequacies of the variables that make up the metabolic syndrome were observed in 21 women with high CA (53.84%), 19 with low HDL-c (48.71%), 13 with high blood pressure (33.33%), 8 had high TG (20.51%) and 3 had high glycemia (7.69%), whereas in the control group the greatest change was in abdominal circumference (50%, n = 16).

**Conclusion:** The frequency of metabolic syndrome was higher in fibromyalgia patients than controls with similar adiposities, with the highest alterations being high CA and low HDL-c.

**Funding:** Coordenação de Aperfeiçoamento de Pessoal de Nível Superior (CAPES) e Conselho Nacional de Desenvolvimento Científico e Tecnológico (CNPq).

**Frequency of metabolic syndrome in adolescents with overweight and sedentary behavior**

N. Leite^1,2^, L. Alle-Furtado^1^, L.V. Tureck^1^, A.C.K. Titski^1,2^, L.M. Brito^1^, P.R.P. Corazza^1^, M.C. Tadiotto^1^, T.A. Biscouto^1^, A. Sulzcach^1^, A.L.K. Souza^1^, M. Czoczuk^1^, D. Andrade^1^, I.C. Jesus^1^, F. Menezes^1^, M.F.A. Lopes^1^, R.B. Radominski^1^, J. Mota^2^

^1^*Federal University of Paraná – Physical Education Departament - Curitiba – Brazil,* ^2^*Porto University – FADEUP - Porto – Portugal*

**Introduction:** Overweight has increased among children and adolescents in the last decades, due to sedentary behavior and inadequate feeding, and may be accompanied by comorbidities. The simultaneous presence of at least three cardiometabolic risk factors compose the diagnosis of metabolic syndrome (MetS), characterizing it as a health problem to be investigated in all age groups.

**Objective:** To verify the frequency of MetS in overweight adolescents with sedentary behavior.

**Methods:** Sixty-four adolescents (26 boys and 38 girls), aged 10 to 16 years, who had a body mass index score-z (IMC-score Z) ≥ 1.5 and sedentary behavior (≥ 2 hours of television per day) and physical activity time (< 60 minutes/day). All were evaluated for height, body mass, waist circumference (WC), systolic (SBP) and diastolic (DBP) blood pressure and body composition (bioimpedance). The maximal oxygen consumption (VO2max) was evaluated by direct method by maximal treadmill test and basal metabolic rate (BMR) by indirect calorimetry. The daily energy expenditure was performed by interview with the three-day recall, considering two days during the week and one day at the weekend. Concentrations of glucose (GLU), total cholesterol (TC), high-density lipoprotein (HDL-C), triglycerides (TG), insulin, total testosterone and S-DHEA were measured. The Homeostasis Model Assessment-insulin resistance (HOMA-IR) and the quantitative insulin sensitivity check index (QUICKI) were determined. Participants were divided according to the presence of MetS, characterized by the presence of three or more of the following criteria, for age and sex: HDL-c < 45 mg/dL; TG > 130 mg/dL; WC > 75 cm; GLU > 100 mg/dL; SBP and/or DBP > 90 mmHg or > 120/80 mmHg. The groups were compared by parametric and non-parametric tests, considering a difference of p < 0.05.

**Results:** The frequency of MetS in the sample studied was 23.44% (n = 15), and the proportion of boys (n = 6) and girls (n = 9) with MetS was similar (*x2* = 0.003, p = 0.807). The group with MetS had higher mean body mass (p < 0.05), height (p < 0.05), fat mass (p < 0.05), fat-free mass (p < 0.001), resting heart rate (p < 0.01), insulin (p < 0.01), HOMA-IR (p < 0.01), TC (p < 0.05) and lower values for QUICKI (p < 0.01). No differences were observed in WC, IMC-score Z and S-DHEA, total testosterone concentrations, as well as in the variables of exercise test (heart rate maximum, VO2max absolute and relative, test time), daily energy expenditure and BMR.

**Conclusion:** MetS was diagnosed in one-quarter of adolescents with overweight and sedentary behavior. Cardiorespiratory fitness and daily energy expenditure were similar among adolescents with and without MetS. The anthropometric measures BMI and WC didn’t differ between groups, being verified greater fat mass in adolescents with MetS, demonstrating that body composition was the differential factor in the diagnosis of MetS between overweight adolescents.

**Funding:** CNPq, CAPES e Ministério da Saúde Edital PPSUS- Fundação Araucária.

**Expression of GLUT1 and GLUT4 in right ventricle in Pulmonary Arterial Hypertension – Effects of chronic treatment with NRG1**

C. Maia-Rocha^1^, R. Adão^1,^ P. Mendes-Ferreira^1^, A.F. Leite-Moreira^1^, C. Brás-Silva^1^

^1^*Departamento de Fisiologia e Cirurgia Cardiotorácica da Faculdade de Medicina da Universidade do Porto, Portugal*

**Introduction:** The healthy heart generates up to 30% of its ATP from glucose. Under conditions of cardiac injury or stress, the heart relies even more heavily on glucose as a source of energy. Glucose is transported into the heart by members of the family of facilitative glucose transporters (GLUTs). The two major isoforms of glucose transporters in the myocardium are GLUT1 and 4. GLUT4 is the predominant GLUT expressed in the adult heart and its genetic ablation in the heart has been shown to result in pronounced cardiac hypertrophy. GLUT1 is a major GLUT expressed in the fetal heart but is increases in abundance in the adult heart in response to myocardial injury or stress^1^.

Neuregulin-1 (NRG1), a protein that has been shown to play beneficial effects on Pulmonary Arterial Hypertension (PAH) and right ventricle (RV) hypertrophy^2^, has also been associated with the regulation of glucose metabolism^3^.

In this study we aimed to investigate the correlation between disease markers and the GLUT1 and 4 expressions in RV, in an animal model of PAH. We also aimed to evaluate the effect of chronic treatment with NRG1 on GLUT1 and 4 expressions in the RV of animals with HF associated with PAH.

**Results:** In this animal model of PAH we found that the decrease in ejection fraction (EF) correlates with increased GLUT1 expression (p = 0.0005) and decrease in GLUT4 expression (p = 0.0167) in RV. We also observed that increased GLUT1 expression correlates with increased hypoxia-inducible factor 1-alpha (HIF1a) expression (p < 0.0001). The decrease in GLUT4 expression was shown to correlate with increased brain natriuretic peptide (BNP) (p = 0.0032) and endothelin 1 (ET1) (p = 0.0006) expression, two markers of overload and hypertrophy.

We observed an increase of GLUT1 RV expression in the MCT group (4.13 ± 0.49AU) compared to the CTRL group (1.00 ± 0.19AU), in the MCT+rhNRG1 group these values were completely reverted (1.66 ± 0.31AU). GLUT4 increased in all groups of animals treated with rhNRG1 (CTRL+rhNRG1 = 1.41 ± 0.09AU and MCT+NRG1 = 1.48 ± 0.18AU vs. CTRL = 1.00 ± 0.16AU and MCT = 0.75 ± 0.02AU).

**Conclusion:** In the present study we observed that the expression of GLUT1 is associated with the development of the disease whereas GLUT4 is affected by chronic treatment with rhNRG1. The expression of GLUT1 and 4 correlate with parameters of cardiac function and with disease markers and chronic treatment with rhNRG1 attenuates the changes in GLUTs induced by MCT. So we can conclude that the therapeutic effects of rhNRG1 in PAH might be due in part to the regulation of GLUT1 and 4 expressions.

**References**

1. Rosenblatt-Velin N, et al. (2001). Postinfarction heart failure in rats is associated with upregulation of GLUT-1 and downregulation of genes of fatty acid metabolism. Cardiovasc Res.Dec;52 (3):407–16.

2. Mendes-Ferreira P (2016) Neuregulin-1 improves right ventricular function and attenuates experimental pulmonary arterial hypertension. Cardiovasc Res.1; 109 (1):44–54.

3. Cantó C, et al. (2004). Neuregulin signaling on glucose transport in muscle cells. J Biol Chem. 279: 260–268.

**Potent pro-apoptotic, cytotoxic, antiproliferative and antimigratory effect of a Catechin: Lysine complex in breast cancer cell lines**

C. Silva^1,2^, A. Correia-Branco^1,2^, P. Sonveaux^3^, J. Stephenne^4^ and F. Martel^1,2^

^1^*Unit of Biochemistry, Department of Biomedicine, Faculty of Medicine, University of Porto, Porto, Portugal*, ^2^*Instituto de Investigação e Inovação em Saúde, University of Porto, Porto, Portugal,* ^3^*Pole of Pharmacology, Institute of Experimental and Clinical Research, Université catholique de Louvain (UCL), Brussels, Belgium,* ^*4*^*BePharBel Manufacturing, Courcelles, Belgium*

Breast cancer is the second most common cancer in the world and the most common cancer among women, both in developed and developing regions^1,2^. In general, polyphenols, abundantly found in plants, display many anticarcinogenic properties, including inhibition of cancer cell proliferation, tumor growth, angiogenesis, metastasis and inflammation, as well as pro-apoptotic effects. Several studies previously indicated that the anticarcinogenic efficacy of polyphenols can be enhanced by combining them with compounds such as amino acids and vitamins^3^. In this context, our study aimed to investigate the effect of a Catechin:Lysine complex (Cat:Lys) on cell proliferation (^3^H-thymidine incorporation assay), growth (sulforhodamine B assay), viability (LDH assay), apoptosis (TUNEL assay) and migration (scratch test) of estrogen receptor-positive human breast cancer cell line MCF7, estrogen receptor-negative human breast cancer cell line MDA-MB-231, and non-cancerous human breast epithelial cell line MCF12A.

MCF7, MDA-MB-231 and MCF12A cells were exposed to Cat:Lys for a short (26 min; 1–5,000 μM) or long (24 h; 1–1,000 μM) period. We report that Cat:Lys (24 h) induces a concentration-dependent decrease in cell proliferation rates, cell growth and cell viability in both MCF7 and MDA-MB-231 cells. In MCF12A cells, a reduction of cell proliferation, growth and viability was observed only with the highest concentrations (0.5–1 mM). Similarly, a more potent effect of Cat:Lys on cell growth and viability was found after short-term exposure (26 min; 1–5,000 μM) in MCF7 and MDA-MB-231 compared to MCF12A cells. Exposure to Cat:Lys (24 h; 0.01 mM or 1 mM) reduced the migratory capacity of MCF7 and MDA-MB-231 cells, but only the highest concentration of Cat:Lys (1 mM) reduced the migratory capacity of MCF12A cells. Moreover, exposure to Cat:Lys (1 mM) for 24 h strongly increased the apoptotic index in MCF7 and MDA-MB-231 cells (to 526 ± 23% and 368 ± 15% of control, respectively), but no significant effect was observed in MCF12A cells. Finally, we verified that the pro-apoptotic effect of Cat:Lys (24 h; 1 mM) in both MCF7 and MDA-MB-231 cells was abolished by simultaneous exposure to a specific inhibitor of the Janus kinase/signal transducer and activator of transcription protein (JAK/STAT) (AG490; 5 μM) or to a specific inhibitor of the WNT pathway (XAV 939; 10 μM).

Taken together, our experimental data show that Cat:Lys has selective anticancer activity. It has very potent antiproliferative, cytotoxic, antimigratory and pro-apoptotic effects in both estrogen-positive and -negative breast cancer cells, but limited effects on a non-cancer epithelial breast cells. Our data indicate that the pro-apoptotic effect of Cat:Lys involves activation of WNT and JAK/STAT signaling. Therefore, Cat:Lys is a strong candidate for the development of a new, effective anticancer agent against breast cancer.

**References**

1. Coughlin SS, Ekwueme DU. *Cancer epidemiology.* 2009;33 (5):315–318.

2. Guedes M, Araújo JR, Correia-Branco A, Gregorio I, Martel F, Keating E. *Experimental cell research.* 2016;341 (2):111–122.

3. Niedzwiecki A, Roomi MW, Kalinovsky T, Rath M. *Nutrients.* 2016;8 (9).

**Funding:** This work supported by BePharBel Manufacturing (Courcelles, Belgium). P.S. is a F.R.S.-FNRS Senior Research Associate.

**The anti-diabetic drug metformin negatively impacts the process of placentation in HTR-8/SVneo cells**

A. Correia-Branco^1,2^, E. Keating^1,3^ and F. Martel^1,2^

^1^*Unit of Biochemistry, Department of Biomedicine, Faculty of Medicine, University of Porto, Porto, Portugal*, ^2^*I3S, Instituto de Investigação e Inovação em Saúde, University of Porto, Porto, Portugal,* ^3^*CINTESIS, Center for Research in Health Technologies and Information Systems, University of Porto, Porto, Portugal*

Placentation is a continuous and highly regulated process performed by extravillous trophoblasts (EVTs), which are fully specialized trophoblasts exhibiting an invasive and proliferative phenotype. EVTs perform the anchorage of the chorionic villi into the uterine wall and actively regulate the process of uterine spiral arteries remodelling, a process that ends up with the establishment of the utero-placental blood flow [1]. Metformin (1,1-dimethylbuguanide; METF) is widely used as first-line treatment in type 2 diabetes and polycystic ovary syndrome (PCOS) [2], being frequently used as therapy in alternative to insulin. METF freely crosses the placenta, thus exposing the fetus to concentrations approaching those in the maternal circulation [2,3]. Nothing is known concerning the effect of METF upon the placentation process.

The aim of this study was to investigate the effect of METF upon placentation-related processes and upon ^3^H-deoxy-D-glucose (^3^H-DG) and ^3^H-folic acid (^3^H-FA) uptake, by a human first-trimester EVT cell line (HTR-8/SVneo cells). Furthermore, involvement of the mammalian target of rapamycin (mTOR), c-Jun N-terminal kinases (JNK) and phosphatidylinositol-4,5-bisphosphate 3-kinase (PI3K) intracellular signaling pathways on the effect of METF was also investigated.

The effect of METF (24 h; 0.01–1 mM) upon placentation-related processes in HTR-8/SVneo cells was determined by quantification of cell viability (lactate dehydrogenase leakage (LDH) assay), proliferation (^3^H-thymidine incorporation assay), culture growth (sulforhodamine B (SRB) assay), migration (wound-healing assay) and apoptosis (TUNEL assay). The effect of METF (24 h; 0.01–1 mM) upon the uptake of glucose and folates was studied by quantification of the cellular incorporation of ^3^H-DG and ^3^H-FA, respectively, by liquid scintillation counting.

A 24h-exposure to METF (1 mM) presented a negative effect upon all the placentation-related processes studied (namely, cell viability, proliferation, culture growth and migration), associated with an increase of the apoptosis index. A lower concentration of METF (0.01 mM), alone or in combination with HG (20 mM), was also able to significantly decrease culture growth and viability. Furthermore, METF concentration-dependently inhibited ^3^H-DG and ^3^H-FA uptake. The inhibitory effect of METF (1 mM) upon cell viability was completely abolished by specific inhibition of mTOR (rapamycin 100 nM), JNK (SP600125 5 μM) or PI3K (LY294002 1 μM), which indicates the involvement of these signaling intracellular pathways in this effect of METF.

We show that METF possesses an antiproliferative, cytotoxic, antimigratory and proapoptotic effect, together with an inhibitory effect in nutrient uptake. As such, our study provides evidence that the widely used anti-diabetic agent METF may present a deleterious impact in the placentation process, which might have long-term consequences upon the developmental programming of metabolic syndrome (MetS) later in life [4].

**References**

1. Ji, L., et al., Mol Aspects Med, 2013. 34 (5): p. 981–1023.

2. Charles, B., et al., Ther Drug Monit, 2006. 28 (1): p. 67–72.

3. Vanky, E., et al., Fertil Steril, 2005. 83 (5): p. 1575–8.

4. Correia-Branco, A., E. Keating, and F. Martel, Reprod Sci, 2015. 22 (2): p. 138–45.

**Acknowledgements and Funding**

We thank Ilda Rodrigues for technical assistance. This work was supported by “Fundação para a Ciência e a Tecnologia” (FCT) and FEDER-COMPETE (Ref UID/BIM/04293/2013 and SFRH/BD/109703/2015).

**Polyphenolic-rich extracts from cooked black bean and cowpea induce cell cycle G1 arrest in HCT116 human colorectal cancer cells independently of p53 status**

C.I. Guedes-Teixeira^1,2^ and C. Pereira-Wilson^1,2,3^

^1^*Department of Biology, University of Minho, University of Minho, 4710–057 Braga, Portugal,* ^2^*CITAB-UM Centre for the Research and Technology of Agro-Environmental and Biological Sciences, University of Minho, 4710–057 Braga, Portugal* ^3^*Centre for Biological Engineering, University of Minho, 4710–057 Braga, Portugal*

Legumes (beans) are a key component of the Mediterranean diet and an important source of protein. Evidences suggest that high consumption of leguminous can help in prevention and management of type II diabetes, cardiovascular diseases and obesity. These effects might be due to the presence of bioactive compounds in the non-digestible fraction including dietary fiber, resistant starch, oligosaccharides and phenolic compounds.

In the present study, we extend the characterization of the health improving effects of beans to the characterization of their anti-colon cancer (CRC) properties. Two species of beans, black (*Phaseolus vulgaris*) and cowpea (*Vigna unguiculata*), have been used and phenolic enriched methanolic extracts made from raw and cooked samples. Effects on viability and cell cycle were tested on isogenic HCT116 cell lines p53 wild-type (HCT116 +/+) and p53-null (HCT116-/-) and compared to the effects of soy extracts prepared in the same way.

Mutations in the tumor suppressor gene TP53 are present in as many as 50% of CRCs and are responsible for deregulation of cell proliferation and apoptosis evasion as well as for resistance to therapeutic agents. It is therefore relevant to find therapeutic and nutritional strategies that benefit the large group of CRC patients that present p53 alterations.

Results show that relative to control and raw bean extracts, the extracts of cooked black and cowpea beans induced significant G0/G1 arrest in both cell lines whereas soy bean extracts were much less active and only on HCT116 +/+ cells. Our data shows that, contrarily to soy, black bean and cowpea may have beneficial effects on CRC patients independently of p53 status.

**Funding:** The authors acknowledge the European Investment Funds by FEDER/COMPETE/POCI – Operacional Competitiveness and Internacionalization Programme, under Project POCI-01–0145-FEDER-006958 and National Funds by FCT - Portuguese Foundation for Science and Technology, under the project UID/AGR/04033/2013.

**Acknowledgements**

CGT acknowledges the financial support provided by the FCT-Portuguese Foundation for Science and Technology (SFRH/BD/52544/2014), under the Doctoral Programme “Agricultural Production Chains – from fork to farm” (PD/00122/2012).

**Chrysin interferes with intestinal fructose absorption and possesses an antioxidant effect – Potential influence on Metabolic Syndrome**

N. Andrade^1^, J.R. Araújo^3^, A. Correia-Branco^1,2^, J.V. Carletti^1^, F. Martel^1,2^

^1^*Unit of Biochemistry, Department of Biomedicine, Faculty of Medicine, University of Porto, Porto, Portugal,* ^2^*I3S, Instituto de Investigação e Inovação em Saúde, University of Porto, Porto, Portugal,* ^3^*Institute Pasteur, INSERM U786, Unity of Molecular Microbial Pathogenesis, Paris 75015, France*

**Introduction:** Metabolic Syndrome (MS) increases the risk for atherosclerotic cardiovascular disease and type 2 Diabetes Mellitus. Fructose consumption has been associated with MS development and a substantial increase in both consumption of this sugar and MS incidence has been observed during the last 30 years^1–4^. Dietary polyphenols have been largely studied due to their human health benefits^5^. Several polyphenols are known to interfere with the intestinal absorption of glucose, but little is known concerning the effect of these phytochemicals on fructose intestinal absorption^6^.

**Aim:** To investigate if the polyphenol chrysin interferes with fructose intestinal absorption and its antioxidant effect.

**Methods:** We tested both the acute (26 min) and the chronic (24 h) effect of chrysin (100 μM) on the uptake of ^14^C-fructose (100 nM) by Caco-2 cells (a cell line mimicking the human intestinal epithelium). Moreover, we tested (by RT-qPCR) the mRNA expression of fructose transporters (GLUT2 and GLUT5) after chronic treatment with this polyphenol (100 μM). Finally, the antioxidant effect of chronic chrysin (24 h; 100 μM) was assessed by quantification of lipid peroxidation (TBARS method).

**Results:** Acutely, ^14^C-fructose uptake was inhibited by chrysin 100 μM (by ± 20%). Chronically, chrysin (100 μM) caused a ± 25% decrease in ^14^C-fructose uptake. Its inhibitory effect was not related to a cytotoxic effect (as determined with the MTT and the LDH assays). GLUT5 is the main carrier involved in the apical uptake of fructose by enterocytes^7^. By using a specific inhibitor of GLUT5 (L-Sorbose-Bn-OZO 10 μM) and phloretin (1 mM), a non-specific inhibitor of GLUTs, we could conclude that chrysin appears to interfere with both GLUT2 and GLUT5. Accordingly, this phytochemical was able to markedly (>80%) decrease the mRNA expression of GLUT2 and GLUT5. Moreover, ^14^C-fructose uptake appears to be dependent on p38 MAPK, PI3K and PKA intracellular signalling pathways, but chrysin does not act through these signalling pathways. Finally, chrysin (100 μM) was able to reduce lipid peroxidation, both in the absence and presence of tert-butyl hydroperoxide (t-BOOH, 500 μM), an oxidative stress inducer.

**Conclusion:** Chrysin was found to be an effective inhibitor of ^14^C-fructose uptake by Caco-2 cells. This compound appears to interfere with both GLUT2 and GLUT5-mediated ^14^C-fructose uptake and it is a stunning inhibitor of GLUT2 and GLUT5 gene expression. This compound possesses also an antioxidant effect. These results suggest that chrysin might decrease the intestinal absorption of fructose, with beneficial effects on type 2 diabetes/MS.

**References**

1. Pereira CD et al. Int. J. Endocrinol. 2014:2014:384583.

2. Elliott S et al. Am. J. Clin. Nutr. 76:911–22; 2002.

3. Rutledge AC, Adeli K. Nutr. Rev. 65:S13–23; 2007.

4. Robert H, Lustig MD. J. Am. Diet Assoc. 110:1307–21; 2010.

5. Vauzour D et al. Nutrients 2:1106–1131; 2010.

6. Araújo J, Martel F. Arq Med. 23:35–43; 2009.

7. Drozdowski LA, Thomson A. World J Gastroenterol. 21:1106–31; 2006.

**Acknowledgements**

Professor Arnaud Tatibouet of the University of Orleans, France, for having provided the inhibitor of GLUT5 and CAPES – Brazilian Federal Agency for Support and Evaluation of Graduate Education within the Ministry of Education of Brazil, for financing this project - PN: 10103/13–9.

**Effects of dietary polyphenol Chrysin upon an animal model of Metabolic Syndrome induced by Fructose**

N. Andrade^1^, I. Rodrigues^1^, S. Andrade^1,2^, C. Silva^1^, E. Keating^1^, F. Martel^1,2^

^1^*Department of Biomedicine, Unit of Biochemistry, Faculty of Medicine, University of Porto, Porto, Portugal,* ^2^*i3S, Instituto de Investigação e Inovação em Saúde, University of Porto, Porto, Portugal*

**Introduction:** Metabolic Syndrome (MS) is a major public health issue worldwide^1^. MS raises the risk of developing cardiovascular disease, type 2 diabetes, non-alcoholic fatty liver disease, cancer, kidney and pancreatic dysfunction^2,3^. Fructose consumption has been associated with MS development and a substantial increase in both consumption of this sugar and MS incidence has been observed during the last 30 years^4–7^. Thus, establishment of appropriate experimental animal models mimicking the effect of fructose consumption in humans is crucial to study MS. Recently,our research team showed that, *in vitro,* the dietary polyphenol chrysin is an effective inhibitor of ^14^C-fructose uptake by human intestinal epithelial cells, interfering with both GLUT2- and GLUT5-mediated fructose uptake and gene expression^8^.

**Aim:** To investigate if chrysin interferes with the development of MS induced by fructose in an animal model.

**Methods:** The study was carried out in 24 adult male CD SDR (220–310 g), from Charles River Laboratories (France). Animals were randomly divided into 4 groups (6 animals each): (A) tap water (Control group), (B) tap water and daily dose of chrysin (100 mg/kg) by oral administration (Chrysin group) (C) 10% fructose in tap water (Fructose group), or (D) 10% fructose in tap water and daily dose of chrysin chrysin (100 mg/kg) by oral administration (Fructose+Chrysin group). All groups were fed *ad libitum* with standard laboratory chow diet and dietary manipulation lasted 18 weeks. Body weight, glycaemia, heart rate, blood pressure and food and fluid intake were registered weekly. After sacrifice, heart, kidneys, liver and epididymal adipose tissue were collected and weighted and a tissue weight/animal weight ratio was calculated.

**Results:** Overall, body weight, heart rate and glycemia revealed similar values in the 4 animal groups. Groups C and D had a significantly lower food ingestion than groups A and B. On the contrary, liquid ingestion was lower in A and B groups than in groups C and D. Of note, group D had a significantly lower food ingestion than group C in the last 3 weeks of treatment. Concerning blood pressure, animals from group A and B had similar systolic and diastolic blood pressure. On the other hand, group C had a significant rise in both systolic and diastolic parameters from week 14^th^ onwards, an increase not observed in group D animals. Finally, the ratio liver/body weight, right kidney/body weight and heart/body weight were significantly changed in group C, in relation to group A. In none of these cases, chrysin seemed to be able to revert the effect induced by fructose.

**Conclusion:** Based on our results, this animal model induced by fructose is able to mimic some of the features of MS. Chrysin seems to have a protective effect on the rise of systolic and diastolic blood pressure induced by fructose-feeding.

**References**

1. Alberti K G et al. Circulation.120:1640–5; 2009.

2. Kaur J. Cardiol Res Pract. 2014:21; 2014.

3. Zimmet P et al. J Atheroscler Thromb. 2005;12 (6):295–300.

4. Pereira, C.D. et al. Int J of Endocrinol. 2014.

5. Elliot, S. et al. Am J Clin Nutr. 76:911–22; 2002.

6. Rutledge, A.C.; AdeliI, K. Nutr Rev. 65:S13–23; 2007.

7. Robert H, Lustig MD. J Am Diet Assoc. 110:1307–21; 2010.

8. Andrade N, et al. J Funct Food. 2017 (in press)

**Acknowledgements**

CAPES – Brazilian Federal Agency for Support and Evaluation of Graduate Education within the Ministry of Education of Brazil, for financing this project - PN: 10103/13–9.

**Statins intolerance: a new approach**

S.A. Machado^1^, P. Barreira^2^, V. Carreiras^3^, T.C. Gonçalves^4^, J. Neves^5^, S. Remtula^4^, R. Sanches^6^, I. Teixeira^7^

^1^*Interna de Medicina Geral e Familiar USF Samora Correia, ACES Estuário do Tejo,* ^2^*Interno de Medicina Geral e Familiar, USF Alcais, ACES Cascais,* ^3^*Médica Assistente na USF Samora Correia ACES, Estuário do Tejo,* ^*4*^*Interno de Medicina Geral e Familiar, USF São Julião, ACES Lisboa Ocidental e Oeiras,* ^*5*^*Interno de Medicina Geral e Familiar, USF Monte Crasto, ACES Gondomar,* ^*6*^*Interno de Medicina Geral e Familiar, USF Conde Oeiras, ACES Lisboa Ocidental e Oeiras,* ^*7*^*Interno de Medicina Geral e Familiar, USF Ribeira Nova, ACES Lisboa Central*

**Introduction and Objectives:** Statins substantially reduce the risk of cardiovascular events. Ten to twenty % of the patients are unable to tolerate statins, which leads to statin discontinuation or low adherence, constituting a problem of great clinical importance [1,2].

A large degree of variability remains as to what is considered to be statin intolerance. There is significant uncertainty regarding its actual incidence and insufficient knowledge or awareness concerning the best therapeutic approaches to the problem. Several reviews have recently been published in order to understand its mechanisms and find alternatives, often with controversial results. Therefore there is no universal consensus or formal recommendations addressing this issue [1–4].

The authors of this paper propose a current definition for statin intolerance, provide tools to identify the population most likely to experience statin-induced adverse effects and recommendations to prevent those, and draw a flow chart of action to approach statin intolerance. Ultimately, alternative and/or new therapies are addressed.

**Methods:** A search was conducted using pubmed-MEDLINE with the key-words ”statin intolerance”. Articles published in the last 5 years written in Portuguese or English with free full-text available were selected. A total of 11 articles were included.

**Results:** Statin intolerance: Statin intolerance can be defined as the occurrence of adverse symptoms (perceived by the patient to be unacceptable), and/or laboratory abnormalities suggesting undue risk attributed to statin therapy. In addition, the degree of statin intolerance can be classified as partial or complete. They include muscular symptoms (myalgia, myopathy, rabdomyolysis), hepatotoxicity, new onset diabetes, and other less frequent [1–5].

Population at risk and External factors associated with statin intolerance: Risk factors for statin intolerance can be analyzed through a detailed history and precautions can be taken. Factors associated with increased risk of statin intolerance can be predicted and sometimes corrected [4–8].

Managing statin intolerance: Individual approach should be prioritized. An analysis on statin choice, scheme and dosage is described in a flow chart of action, including instructions concerning solution or clarification of adverse symptoms or laboratorial abnormalities.

Statin maintenance should always be preferred, even if a lower dose or different statin. Other lipid-lowering drugs may be needed to achieve the appropriate targets, in combination or in substitution to the statin. Clarifications regarding other therapies and a debate on new strategies to control lipid profile, such as HDL therapies (vitamin B3 or niacin), triglyceride therapies (fibrates) or new therapies, like ANGPTL3 inhibitors, mipomersen, lomitapide or PCSK9 inhibitors [1,2,5,6,8–12].

**Conclusion:** Given the profound cardiovascular benefits of statins, it remains the mainstay of lipid-lowering treatment for most of the patients. Establishing a correct diagnosis of statin intolerance is therefore of at most importance, and an adequate therapeutic approach is more complex than simple discontinuation of statin therapy. New therapies are emerging and promising a valuable help achieving cardiovascular protection in these patients.

**References**

1. Tomáš Stul, et al, Curr Atheroscler Rep (2015) 17: 69.

2. Michael D. Shapiro, et al, Circ Res (2016) 118: 732–749.

3. Robert S Rosenson, et al, Cardiovasc Drugs Ther (2017) 31:179–186.

4. Maciej Banach, et al, Arch Med Sci (2015) 11: 1–23.

5. Anna Gluba-Brzozka, et al, Arch Med Sci (2016) 12: 645–658; David H. Fitchett, et al, Circulation (2015) 131: e389–e391.

6. Soma B Raju, et al, Indian J Endocrinol Metab (2013) 17: 977–982; Warner M. Mampuya, et al, Am Heart J (2013) 166: 597–603.

7. Travis Y. Morioka, et al, Atherosclerosis (2015) 238: 77–82.

8. Rafael Bitzur, et al, Diabetes Care (2013) 36: S325–330.

9. Michele M. Gulizia, et al, Eur Heart J Suppl (2017) 19: D55–D63.

10. Erik S. Stroes, et al, Eur Heart J (2015) 36: 1012–1022.

11. Norman E. Lepor, et al, Am Health Drug Benefits (2015) 8: 483–489.

12. Carl E. Orringer, et al, J Clin Lipidol (2017) 11: 880–890.

**Effect of DDE on glucose and fatty acid uptake in hepatocytes**

P. Soares^1^, D. Sousa^1^, M. Cruz^1^, F. Pego^1^, E. Keating^1,2^ and R. Negrão^1,3^

^1^*Unit of Biochemistry, Department of Biomedicine, Faculty of Medicine, University of Porto, Porto, Portugal,* ^2^*CINTESIS - Center for Health Technology and Services Research, ProNutri - Clinical Nutrition & Disease Programming, Porto, Portugal,* ^3^*I3S, Instituto de Investigação e Inovação em Saúde, University of Porto, Porto, Portugal*

**Introduction:** Despite the legislation restricting their use, persistent organic pollutants, such as p,p’-dichlorodiphenyldichloroethylene (DDE), still persist in the environment with detectable levels in humans, particularly in adipose tissue. Several studies have identified a possible relationship between DDE accumulation and obesity, metabolic syndrome and type-2 diabetes. However, the mechanisms underlying this relationship remain poorly understood.

**Objective:** To verify the influence of DDE exposure on glucose and fatty acid uptake, on *GLUT1*, *FATP4* and *ACSL1* gene expression and on lipid accumulation by hepatocytes (Huh-7 cells).

**Results:** DDE did not change hepatocyte glucose uptake (^3^H-desoxy-D-glucose) or *Glut1* expression (qRT-PCR). Nevertheless, it decreased lipid accumulation (Oil-Red-O) in a process dependent on glucose concentration (higher glucose concentration, lower lipid accumulation).

DDE seemed to increase hepatocyte fatty acid uptake (Abcam Kit for Free Fatty Acid Uptake Assay) and *FATP4* and *ACSL1* expression (qRT-PCR), but lipid accumulation decreased (Oil-Red-O) after exposure to fatty acids.

**Conclusion:** DDE interfered with lipid metabolism in Huh-7 cells but the mechanism underlying this effect must be further investigated to better understand the association of this pollutant with metabolic syndrome.

**Funding:** This study was partially funded by Fundação para a Ciência e Tecnologia, Portugal (FCT) (UID/BIM/04293/2013).

**Is low dose radiation exposure a risk factor for atherosclerotic disease?**

P. Boaventura^1,2∗^, C. Durães^1,2∗^, A. Mendes^1,2^, N. Rios Costa^1,2^, I. Chora^3^, S. Ferreira^3^, E. Araújo^3^, P. Lopes^3^, G. Rosa^3^, P. Marques^3^, S. Tavares^3^, V. Chaves^3^, P Bettencourt^3,4^, I. Oliveira^3^, F. Costa^3^, I. Ramos^3,4^, M.J. Teles^3,4^, J.T. Guimarães^3,4^, M. Sobrinho-Simões^1,2,3,4^, P. Soares^1,2,4^^∗^These authors contributed equally to this work

^1^*Ipatimup - Institute of Molecular Pathology and Immunology of the University of Porto, Rua Júlio Amaral de Carvalho 45, 4200–135 Porto, Portugal*, ^2^*i3S - Instituto de Investigação e Inovação em Saúde, Universidade do Porto, Rua Alfredo Allen 208, 4200–135 Porto, Portugal,* ^3^*S. João Hospital, Alameda Prof. Hernani Monteiro, 4200–319 Porto, Portugal,* ^4^*Faculty of Medicine of the University of Porto, Alameda Prof. Hernani Monteiro, 4200–319 Porto, Portugal*

Non-targeted late effects of radiation exposure include an increased risk of cardiovascular disease which is still debatable in the low dose radiation context. Tinea capitis patients treated in childhood with x-rays to induce scalp epilation received a low radiation dose in their carotids. Aiming to better clarify this issue, we evaluated carotid atherosclerosis in a cohort of such patients treated in 1950–1963 in Portugal.

A group of 454 individuals randomly chosen from previously observed Portuguese tinea capitis patients, and a control group mainly composed by their spouses (n = 280), were enrolled. Cardiovascular risk factors such as waist circumference, weight, BMI, blood pressure, tobacco consumption, and biochemical measurements were obtained. Ultrasound imaging of carotid arteries for intima media thickness and stenosis evaluation was performed according to a standardized protocol.

Irradiated individuals were significantly older, more frequently never smokers, hypertensive, and presented higher glycated haemoglobin and alkaline phosphatase levels, in comparison with the non-irradiated individuals. The irradiated group showed a higher frequency of ≥30% carotid stenosis than the non-irradiated group (13.9% *vs* 10.7%) but this difference was not statistically significant (p = 0.203). The percentage of patients with ≥50% stenosis was 2.9% in the irradiated group and 0.4% in the non-irradiated group (p = 0.016). The statistical significance was retained after adjustment for confounding variables (p = 0.039), but our analysis had not sufficient statistical power (<80%) due to the small number of cases presenting a degree of stenosis ≥50%. Moreover, no significant differences were observed in carotid intima-media thickness between the two groups. The present study does not confirm low dose radiation exposure as a risk factor for carotid atherosclerotic disease.

**Homology modeling and molecular docking of the structural determinants of Janus kinase 3 and its interaction with b-catenin**

Jugal Kishore Das, Jayshree Mishra, Narendra Kumar

Department of Pharmaceutical1 Sciences, ILR College of Pharmacy Texas A &M Health Science Center Kingsville TX 78363, USA.

*E-mail address:* *nkumar@pharmacy.tamhsc.edu* *(Narendra Kumar)*

Janus kinase 3 (Jak3) is a non-receptor tyrosine kinase which plays a key role in regulating chronic low grade inflammation mediated Metabolic Syndrome. Previously, we have shown that loss of Jak3 mediated phosphorylation of β-catenin leads to compromise in Adherens Junction (AJ) and facilitation of epithelial mesenchymal transitions (1).

The structural modifications induced in β-catenin due to its phosphorylation by Jak3 and their functional implications in regulating epithelial mesenchymal transitions (EMT) in AJ are unknown. Therefore, in this report, we used in-silico molecular modeling and simulations approach to gain deeper insights into the dynamic interplay between Jak3, β-catenin and E-cadherin in maintaining the integrity of the AJ. In order to simulate the impact of phosphorylation of tyrosine residues (Y30, Y64, and Y86, and Y654) of β--catenin, these amino acids were substituted with the phosphomimetic residue Glutamic acid (E). The conformational changes induced in the structure of β-catenin were analysed by superimposition and energy minimization using CHARMM Forcefield and visualized in Accelerys Discovery Studios (Biovia). Further, β-Catenin-Jak3 interactions were studied by using protein-protein docking by GRAMMx docking software.

We found that EGF mediated phosphorylation of Y654 induced conformational changes in β-Catenin inhibited its interaction with E-cadherin in the AJ. The structural changes were reversed by Jak3 mediated phosphorylation of Y30, Y64 and Y86 increasing the affinity of β-Catenin- E-Cadherin interactions in the AJ. Furthermore, we found that the FERM domain of Jak3 interacted with the NTD of β-Catenin facilitating the juxtaposition of indicated tyrosine residues of β-Catenin with the kinase domain of Jak3 thereby validating Jak3-mediated phosphorylation of β-Catenin.

Understanding the regulation of Jak3 activation and its phosphorylation of β-catenin protein in patients with metabolic syndrome and associated cancer-metastasis, may lead to development of efficient strategies to inhibit EMT.

**Keywords:** Jak3, β- catenin, Adherence Junction, Epithelial- Mesenchymal Transitions.

**Reference**

1. Jayshree Mishra, Jugal Kishore Das and Narendra Kumar. Janus kinase 3 regulates adherens junctions and epithelial mesenchymal transition through β-catenin J. Biol. Chem. 2017 292: 16406–16419.

**Sponsorship:** These studies were supported by grants from Crohn's & Colitis Foundation of America (CCFA Ref # 2188) and NIH (DK081661) to NK and National Institute of Health-SBIR (GM109528) to JM and NK.

